# Serological diagnosis and prevalence of HIV-1 infection in Russian metropolitan areas

**DOI:** 10.1186/s12879-020-05695-z

**Published:** 2021-01-07

**Authors:** D. E. Kireev, V. P. Chulanov, G. A. Shipulin, A. V. Semenov, E. V. Tivanova, N. M. Kolyasnikova, E. B. Zueva, V. V. Pokrovskiy, C. Galli

**Affiliations:** 1 Federal Budget Institute of Science Central Research Institute of Epidemiology Federal Service for Surveillance on Consumer Rights Protection and Human Wellbeing (Rospotrebnadzor), Novogireyevskaya St., 3A, 111123 Moscow, Russia; 2 I.M. Sechenov First Moscow State Medical University, Moscow, Russian Federation; 3 Center of Strategical Planning and Management of Biomedical Health Risks of the Ministry of Health, Moscow, Russia; 4 St. Petersburg Pasteur Research Institute of Epidemiology and Microbiology, St. Petersburg, Russia; 5 North-Western State Medical University named after I.I. Mechnikov, St. Petersburg, Russia; 6 Abbott Diagnostics, Rome, Italy

**Keywords:** HIV infection, Epidemiology, Recent infections, High risk groups, 4th generation HIV immunoassays, Assay performance

## Abstract

**Background:**

HIV infection is a major health problem in Russia. We aimed to assess HIV prevalence in different population groups and to compare the characteristics of 4th generation immunoassays from Abbott, Bio-Rad, Vector-Best, Diagnostic Systems, and Medical Biological Unit.

**Methods:**

The study included 4452 individuals from the general population (GP), 391 subjects at high risk of HIV infection (HR) and 699 with potentially interfering conditions. HIV positivity was confirmed by immunoblot and by HIV RNA, seroconversion and virus diversity panels were also used. HIV avidity was employed to assess recent infections.

**Results:**

The prevalence in GP was 0.40%, higher in males (0.62%) and in people aged < 40 years (0.58%). Patients attending dermo-venereal centers and drug users had a high prevalence (34.1 and 58.8%). Recent infections were diagnosed in 20% of GP and in 4.2% of HR. Assay sensitivity was 100% except for one false negative (99,54%, MBU). Specificity was 99.58–99.89% overall, but as low as 93.26% on HR (Vector-Best). Small differences on early seroconversion were recorded. Only the Abbott assay detected all samples on the viral diversity panel.

**Conclusion:**

HIV infection rate in the high-risk groups suggests that awareness and screening campaigns should be enhanced. Fourth generation assays are adequate but performance differences must be considered.

**Supplementary Information:**

The online version contains supplementary material available at 10.1186/s12879-020-05695-z.

## Background

Despite progresses towards the WHO/UNAIDS 90–90-90 goals [1] the HIV infection pandemic has not stopped and the countries of Eastern Europe and Central Asia, including Russia, report an increasing number of newly diagnosed cases of infection [2]. The most recent data available for RUSSIA & CIS (Commonwealth of Independent States) indicate that in 2017 more than 33 million HIV tests were performed [3, 4] and the estimate number of new HIV diagnoses exceeded 100,000 [3]. Prevalence and incidence as of December 31, 2017 in the Russian Federation amounted to 664.8 and 72.8 per 100 thousand population, respectively. In 2017, 54.2% of new cases of infection were associated with heterosexual transmission, 42.9% - parenteral drug usage and 2.1% - homosexual transmission [4]. Due to the stigma of homosexual orientation in the country suggests that in reality, cases of infection among MSM are under-reported, and the actual frequency of this route of transmission may be much higher.

Timely detection of HIV-infected individuals is an effective way to combat the pandemic spread. HIV incidence may be reduced by the implementation of large scale screening in the general population and increasing the access to testing for high risk core groups. In both instances, screening shall be performed by high sensitive assays that enable to diagnose the infection in the early stages, when the replicative activity of HIV is higher and likelihood to transmit the infection is greater [5]. On this purpose, in the Russian Federation as well as in most countries testing for HIV infection is performed using the 4th generation HIV immunoassays that simultaneously detect anti-HIV-1 and/or anti-HIV-2 antibodies and the *gag* antigens p25 or p24 [2, 6]. Besides early detection, an additional challenge in the diagnosis is imposed by extreme variability of the human immunodeficiency virus. Within one HIV subtype, virus heterogeneity is 8 to 17%, but it can reach 42% between different subtypes [7], naturally leading to different antigenic structure of the virus and, hence, hampering the efficacy of diagnostic tests. In Russia, a long list of ELISA/CLIA tests for the laboratory diagnosis of HIV infection has been registered and can now be used. However, the available information on their operational characteristics is often limited. Scarce publications either describe the efficacy of various test systems [8, 9] or compare between them [10–12]]. The purposes of this study were to evaluate the prevalence of HIV infection in a sample of the general population and in subjects belonging to high risk groups and to compare the performance characteristics on those populations, on other groups of patients and on special panels of several HIV 4th generation test systems used in Russia.

## Methods

### Clinical specimens

The aim was to enroll 4500 subjects representative of the general population and 1000 subjects from key groups, either patients with potentially interfering conditions (pregnancy, systemic autoimmune diseases, hemodialysis) or at high risk of infection, including injecting drug users and patients with or at risk for sexually transmitted infections followed at a dermo-venereal clinics. All patients were 18 years old or higher and in a fasting status since at least 4 h and were consecutively enrolled among those who accessed the diagnostic centers of the Central Research Institute of Epidemiology (CRIE, Moscow) and Pasteur Research Institute of Epidemiology and Microbiology (Pasteur, St. Petersburg) to perform routine biochemistry analyses for non-infectious indications. All patients enrolled signed an informed consent form and the study was approved by ethics committees of both institutes. Samples were anonymized by a numeric code and the only demographic information recorded were sex and age. A single serum sample from each subject was collected in BD Vacutainer SST II Advance tubes with serum separator. After centrifugation (1500-2000 g, for 20 min at room temperature), each serum was divided in five aliquots of 1 mL each into Eppendorf tubes and stored at ≤ − 20 °C. Specimens were transported at the same temperature to CRIE, where all testing was performed.

#### Control panels

The analytical characteristics of the compared test systems were also evaluated using nine ZeptoMetrix Corporation seroconversion panels (cat. No. 6248, 9012, 9014, 9018, 9021, 9023, 9028, 9031, 9089) and the HIV-1 diversity panel, Ag p24 (R&D, Abbott Diagnostics), containing a fixed amount of HIV-1 p24 antigen from different HIV-1 groups and subtypes.

### HIV 4th generation test systems

All clinical samples and control panels were analyzed in parallel by the following test systems: Architect HIV Ag/Ab Combo (cat. No. 4 J2732, Abbott Laboratories, Wiesbaden, Germany, hereinafter Abbott); Genscreen Ultra HIV Ag-Ab (cat. No. 72386, Bio-Rad, France, hereinafter Bio-Rad); CombiBest HIV-1,2 AG/AT (cat. No. D-0152, Vector-Best, Russia, hereinafter Vector-Best); DS-EIA-HIV-AGAB-SCREEN (cat. No. I-1654, Diagnostic Systems, Russia, hereinafter DS); HIV-1,2-AG/AT (cat. No. IP-113-20, Medical Biological Union, Russia, hereinafter MBU).

### Testing algorithm and interpretation of the results

Each serum specimen was analyzed in parallel by the 5 test systems. According to the assay specifications, initial reactive samples i.e. yielding a sample to cutoff (S/CO) ratio > 1.00, were tested again in duplicate by the same assay. The HIV positivity on samples yielding a repeat reactivity (RR) by any assay was confirmed by the immunoblot assay (IB) INNO-LIA HIV I/II Score (cat. No. 80540, Fujirebio, Japan). Whenever IB yielded a negative or indeterminate result, a second aliquot from the same sample was assayed for HIV-RNA by the RealBest RNA HIV (cat. No. D-0198, Vector-Best, Russia), with a sensitivity of 20 IU/mL and classified as true or false positive according to a positive or negative result by that assay. Testing algorithm and scheme of the confirmatory HIV testing are depicted in Supplemental Figs. 2 and 3. Sensitivity and specificity of the five assays were calculated according to the final classification as true positive, false positive, true negative and false negative and evaluated on the whole study population as well as on different subgroups.

### HIV avidity

An experimental method for assessing antibody avidity has been used on samples confirmed positive for HIV antibodies. This method requires a pretreatment of serum samples and testing by the ARCHITECT HIV assay, has been previously described in detail [13, 14] and allows to discriminate accurately recent (supposed infection date < 6 months before testing) HIV infections. The results are expressed as avidity index value (AI), with indexes < 0.80 indicating a recent infection whereas an AI > 0.80 suggests an established infection, at any disease stage. To account for the imprecision of the method [13, 14] AI results between 0.75 and 0.84 are classified as gray zone.

### Data analysis

Sensitivity, specificity and overall accuracy of the screening assays vs. the final classification as HIV positive or negative have been evaluated by 2 × 2 contingency tables, with a confidence interval (CI) set at 95%. The significance of differences between percentages was evaluated by chi square. The signal to cutoff ratio (S/CO) on negative results has been plotted and the distribution has been evaluated as mean, standard deviation (SD) and SD ratio, expressed as the number of SD from assay cutoff and the negative sample mean by the formula: 1.00 – assay mean S/CO / SD.

## Results

### Study population and HIV prevalence

Enrollment took place between May 2017 and March 2018 and a total of 5586 subjects were initially included. One hundred forty-four were excluded, of which 130 did not meet the inclusion/exclusion criteria (age, fasting), 7 did not have sufficient serum for all tests, 3 recalled the informed consent and 4 were enrolled twice. Testing and data analysis were then performed on 5442 specimens. Of those, 4452 came from the general population (GP), 160 were from pregnant women (PW), 232 from hemodialysis patients (HD), 207 from patients with autoimmune diseases (AU), 262 from drug users (DA), 129 from patients of sexual transmitted diseases clinics (STD). Further details on study populations are provided in Table 1.

**Table 1:** Demographic characteristics of the study population

Patient group	N	Males (%)	Females (%)	Age mean + s.d. (median)	Age range
General population	4452	1693 (38.0)	2759 (62.0)	38 + 10.5 (36)	18–65
Pregnant women	160	0 (−)	160 (100)	30 + 4.5 (30)	20–42
Hemodialyzed	207	116 (50.0)	116 (50.0)	53 + 10.8 (55.5)	20–65
Autoimmune diseases	232	52 (22.4)	157 (77.6)	46 + 13.9 (49)	18–65
Drug users	262	229 (87.4)	33 (12.6)	36 + 8.0 (35.5)	18–57
STD patients	129	59 (45.7)	70 (54.3)	35 + 9.0 (32)	18–63

*s.d.* standard deviation, *STD* sexually transmitted diseases

**Fig. 1:**
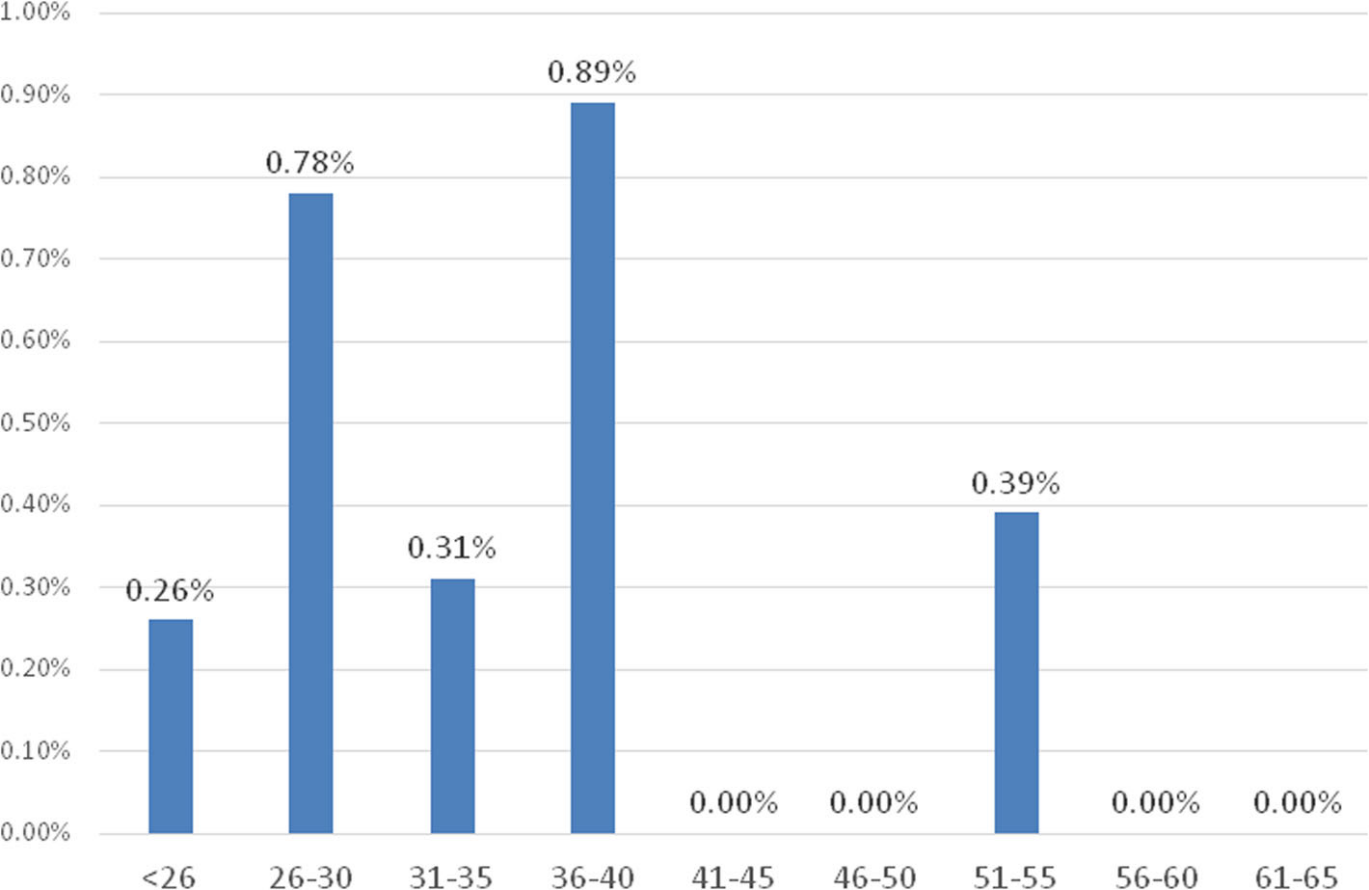
Prevalence of HIV positivity in 4452 subjects from the general population by age groups. Subjects aged < 41 years had a prevalence of 0.58% compared to 0.05% in older subjects

Overall, 217 subjects (3.99%) were confirmed positive for HIV. Of these, 18 were detected in the general population (0.40%), 198 in the two high-risk cohorts (50.64%), and 1 in the cohort of individuals with potentially interfering conditions (0.17%). In the general population the prevalence was significantly higher among males (0.62% vs. 0.17% among females; *P* < 0.001) and the frequency by age groups showed a trend to increase until 40 years of age (Fig. 1), being much higher in people below that age (0.58%) compared to people aged > 40 years (0.05%; *p* < 0.001). HIV positivity was absent among PW and AU and just one HIV case was found among hemodialysis patients (Fig. 1). On the other side, the rate of infection was highest among DA (58.8%) and very high also among STD patients (34.1%). HIV antibody avidity could be assessed on 203/217 HIV positives (92.7%), 15 from GP and 188 from HR. On 5 samples (2.4%, all HR) the result was in the gray zone and time of infection was not established. On the remaining 198, the rates of recent infections were 20.0% in GP, 4.3% among patients at high risk (*p* < 0.01) and 5.4% overall.

### Assay sensitivity and specificity

Four of the HIV 4th generation immunoassays detected all positive specimens, the exception being HIV-1,2-AG/AT (MBU) which failed to find one positive specimen in the general population and had thus a diagnostic sensitivity of 99.54%. The sample from this subject yielded a S/CO ratio lower than the ones attained on the other positive samples on all other four screening assays, and was scored as indeterminate by IB (single weak band corresponding to the p31 protein). The presence of infection in this patient was confirmed by HIV-RNA PCR, and this case was deemed as an acute infection in Fiebig stage II [15].

As for specificity, all assays gave some false positive results. The highest frequency of false positives (22 cases) was shown by Vector-Best and a significant number of such results (13 cases) were identified among drug users from a high-risk cohort. For this cohort, the specificity was 96.37% by Bio-Rad and 93.26% by Vector-Best, and this led the latter to yield the lowest specificity and accuracy among those five studied (Table 2). The specificity of other test systems varied from 99.71% (DS) to 99.89% (MBU).

**Table 2:** Specificity and overall accuracy (rate of correct identification of true negatives and true positives) of the five 4th generation HIV assays

Test system	Specificity – all	Specificity - GP	Specificity – high risk	Specificity – potential interference	Accuracy
Abbott	99.85%	99.84%	99.48%	100%	99.85%
Bio-Rad	99.71%	99.89%	**96.37%**	99.50%	99.72%
Vector-Best	99.58%	99.93%	**93.26%**	99.00%	99.60%
DS	99.77%	99.77%	99.48%	99.83%	99.78%
MBU	99.89%	99.91%	99.48%	99.83%	99.87%

*GP* general population. Bold figures indicate a significantly lower specificity, below the threshold recommended by WHO

Accuracy of all 5 test systems, which in this case characterizes the overall ability to correctly identify all positive and all negative specimens, was higher than 99.50% and varied from 99.60% (Vector-Best) to 99.87% (MBU). Detailed results are presented in Table 2.

### Incidence of initial and repeated positive results, reliability of negative and positive results

According to current regulations in Russia [3], repeated double testing of serum specimens should be performed using the same test system which yielded an initial reactive result. Only Architect HIV Ag/Ab Combo test system showed 100% convergence between the first and second test results. Other systems did not always confirm initial positive result. The convergence was significantly lower in the general population cohort, where the difference between the initial positive and repeated positive specimens reached 44.74% (MBU). In the cohort including individuals at high risk or with potentially interfering conditions, the difference between the initial positive and repeated positive results was less and varied from 0% (Abbott) to 6.04% (Vector-Best). In general, initial positive results were most rarely confirmed when the CombiBest HIV-1,2 AG/AT test system was used (10.49%). Detailed results are presented in Supplemental Table 1.

Reliability of negative and positive results was also determined from the signal-to cutoff (S/CO) ratios. Values of this parameter are known to be proportional to the number of antigen-antibody complexes formed during the reaction, and the ratio near the cut-off value is associated with higher probability of discordant results of repeated testing. In the Abbott test system, lower standard deviation and higher SD/cut-off ratio were obtained and this contributes to the 100% convergence between initial positive and repeated positive results, which was demonstrated by this test system. By comparison, the Vector-Best test system had a comparable SD/cut-off ratio but the highest SD value and lowest number of SD to cut-off among the systems under comparison and this may be the main reason for low specificity and weak convergence between the initial positive and repeated positive specimens. The S/CO distribution results by all assays are reported in Table 3, and the direct comparison between Abbott and Vector-Best is depicted in Supplemental Fig. 1.

**Table 3:** Analysis of the distribution of negative results by five HIV Combo assays. Signal-to-cutoff (S/CO) ratios, standard deviation (SD) and SD ratio from the mean S/CO to the cut-off by the five HIV 4th generation screening assays employed

Test system	Number of specimens	Mean S/CO value	Standard deviation (SD)	SD to cut-off ratio
Abbott	5217	0.11	0.048	18.443
Bio-Rad	5210	0.32	0.118	5.777
Vector-Best	5203	0.13	0.139	6.310
DS	5213	0.09	0.1	9.134
MBU	5220	0.12	0.136	6.493

### Seroconversion panels and virus diversity panel

**Fig. 2:**
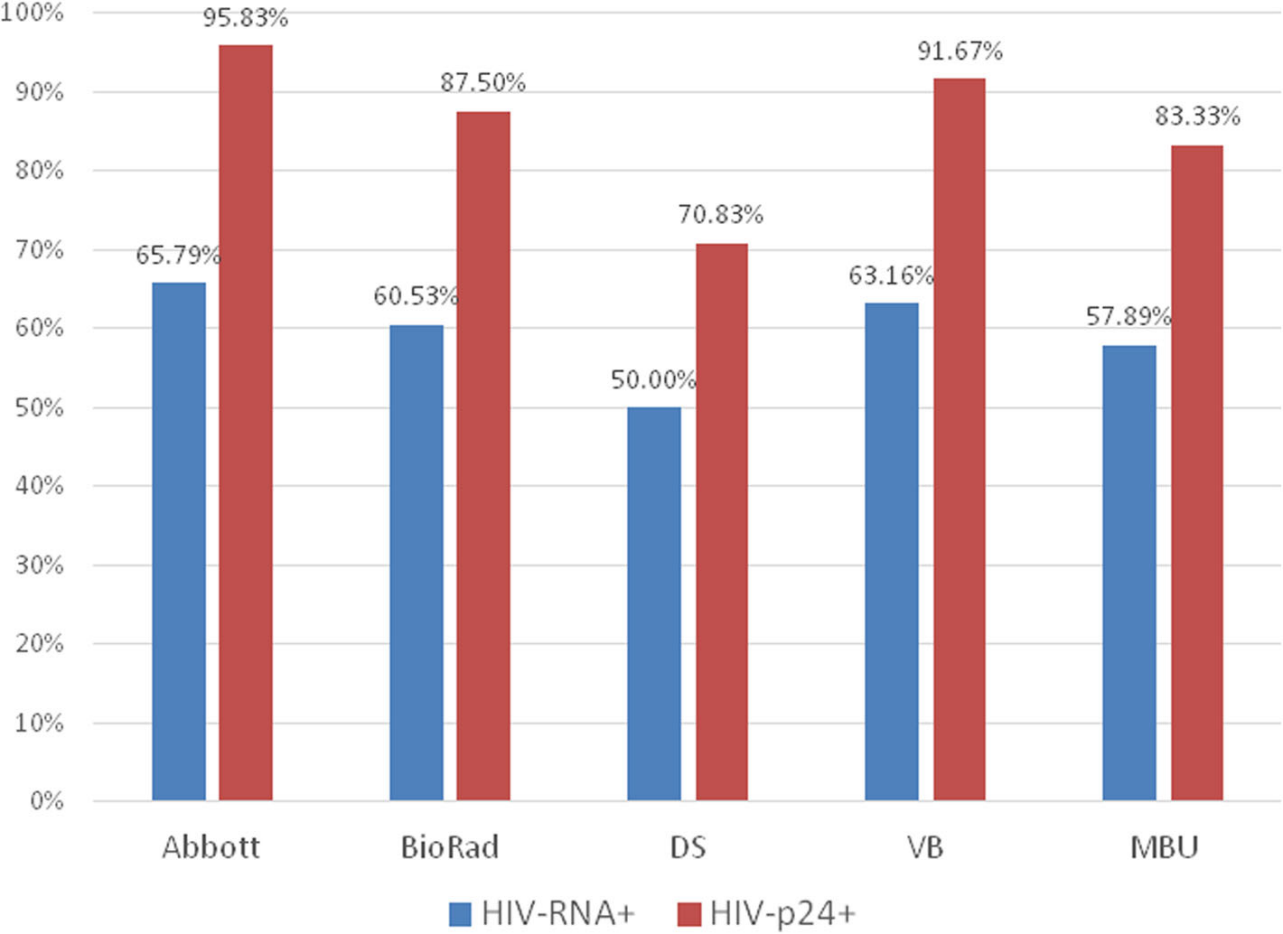
Positivity rates by five HIV 4th generation assays compared to the presence of HIV-RNA and of p24 antigen on 108 samples from nine HIV-1 seroconversion panels (Zeptometrix)

The 9 seroconversion panels included a total of 104 specimens. According to the manufacturer’s package inserts, HIV RNA was detected in 38 of those and p24 antigen in 24 specimens. After testing by the five HIV screening assays, the greatest number of positive results was obtained using the Architect HIV Ag/Ab Combo test system (29 specimens), and the smallest with the DS-EIA-HIV-AGAB-SCREEN system (23 specimens). The time difference between an initial positivity for HIV-RNA and the first reactive result by the most sensitive immunoassay (Abbott) had a mean of 4.50 + 2.45 days and a median on 3.50 days. The results obtained by the five screening assays compared to the positivity for HIV-RNA and p24 antigen are presented in detail in Fig. 2.

**Fig. 3:**
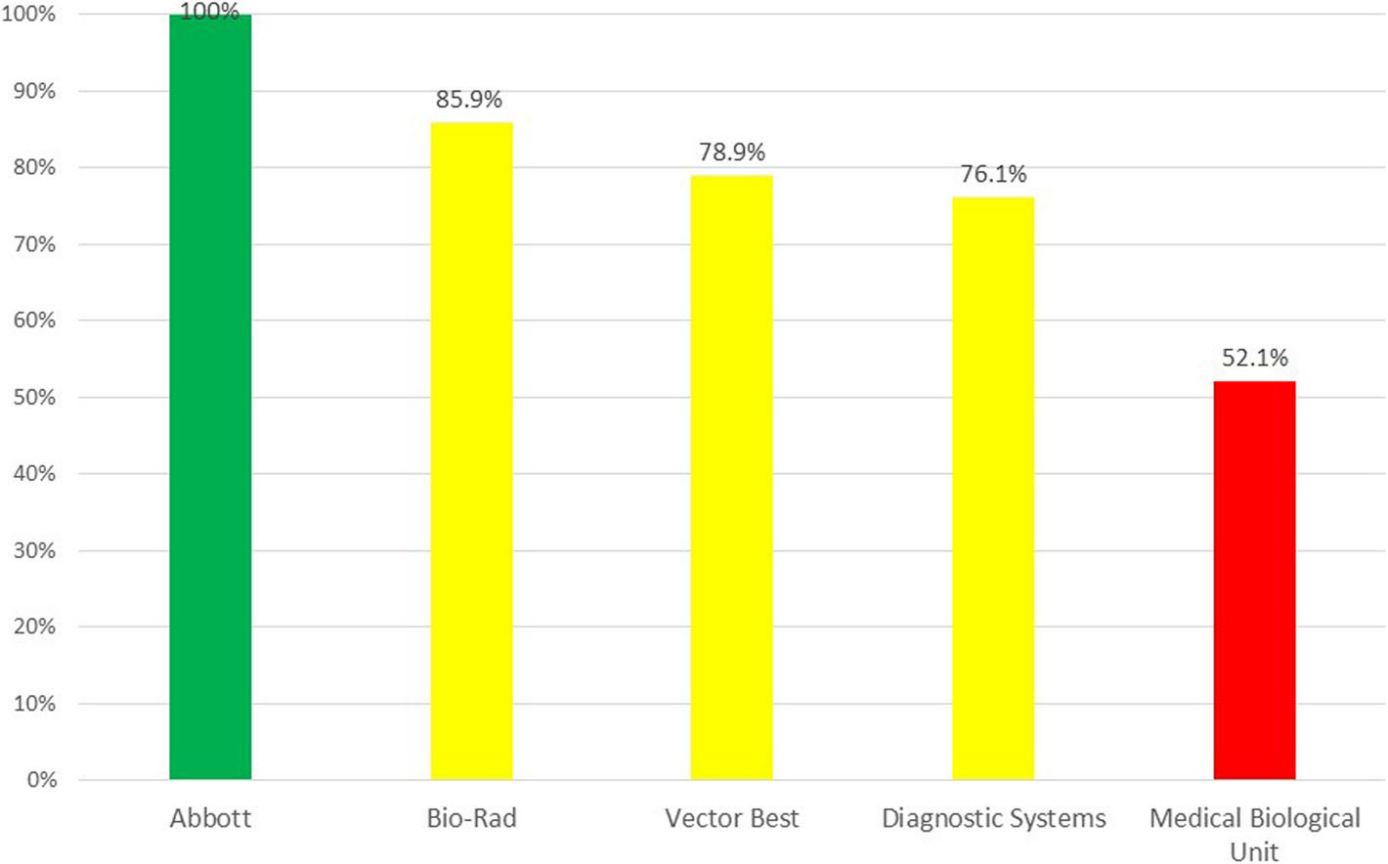
Positivity rates by five HIV 4th generation assays on a HIV-1 p24 viral diversity panel. Green, yellow and red bars indicate an optimal, fair to good and insufficient performance, respectively

Finally, the results of the viral diversity panel study demonstrated a significant difference between the test systems in their ability to detect different HIV-1 subtypes. Not only rare HIV-1 groups were detected with expectedly low efficacy, but the same was true for the specimens containing HIV-1 group M. All specimens of the panel were detected only by the Abbott test system, while the lowest efficacy was demonstrated by the MBU test system (52.1%, 37 out of 71). In general, the three test systems manufactured in Russia (Vector-Best, Diagnostic Systems and Medical Biological Unit) showed a poorer performance (Fig. 3). Detailed results of the panel testing are presented in Supplemental Table 2.

## Discussion

Testing for HIV infection allows for both primary and secondary prevention and is of paramount importance to reduce the burden of this infection and to eventually reach the goals set for 2030 (90% of people with HIV diagnoses, 90% of those diagnosed linked to care, 90% of viral suppression among the treated individuals) [1]. In 2010, the FDA approved the first 4th generation antigen (Ag)/antibody (Ab) combination assay that can detect HIV p24 antigen, detectable in blood plasma within a week after HIV RNA can first be detected [15, 16]. This approval led to updated recommendations from the Centers for Disease Control and Prevention [5] to use Ag/Ab combination assays for initial HIV screening and this approach has been adopted also in the Russian Federation. However, we have only scarce information about the testing systems available in Russia, especially when it comes to domestic manufacturers. This prompted us to compare five 4th ELISA/CLIA test systems used in Russia for the diagnosis of HIV infection on a quite large number of clinical specimens both from the general population and from the groups at risk, as well as on seroconversion and virus diversity panels for a more comprehensive appraisal of assay performances.

According to the World Health Organization document describing the diagnostic characteristics of serological tests used to detect HIV infection, the minimum acceptable parameters for screening tests are 100 and 98% for sensitivity and specificity, respectively [17]. The results of this study are similar to previous studies on the assessment of diagnostic sensitivity and specificity of foreign test systems [18, 19]. However, the desired level of sensitivity was apparently not attained by one of the assays we considered (HIV-1,2-AG/AT manufactured by the Medical Biological Union), who missed an early HIV positivity on a sample from acutely infected subject (Fiebig II stage) [15, 16]. Also, the CombiBest HIV-1,2 AG/AT test system has the lowest specificity on clinical samples.

As the HIV epidemic in the Russian Federation continues to grow, the increased heterogeneity of the viral population and the emergence of new unique forms of the virus are registered. It is also noteworthy to mention numerous peculiarities of the circulation of HIV-1 variants in the Russian Federation as compared to that in the Western Europe, America, Africa, and Asia. In early 2000s, HIV-1 subtype A, currently classified as subtype A6, was responsible for > 90% new infections. As of now, it is spread among > 80% of HIV-infected people. Subtype B HIV-1 is found in approximately 8% patients. Less common are subtypes G, C, D, recombinant forms CRF01_AE, CRF02_AG, CRF06_cpx, CRF11_cpx, CRF63_02A1, and AB-recombinants [20, 21]. Such variability may negatively affect the analytical sensitivity of the tests, as demonstrated in different studies [22–24]. Virus sequencing in detected positive samples was not carried out in accordance with the protocol, therefore it is impossible to say what was the distribution of subtypes in the studied cohort. In view of this, it was important to assess the ability of available test systems to detect HIV from different groups and for this purpose, we used an HIV-1 Virus Diversity panel [24] containing not only HIV-1 group M, but also viruses of such rare groups as N, O, and P. The results of the panel study demonstrated a significant difference between the test systems in their ability to detect different HIV-1 subtypes. Moreover, not only rare HIV-1 groups were detected with expectedly low efficacy, but the same was true for the specimens containing HIV-1 group M, namely A and B. The results of virus diversity panel testing by the Medical Biological Union product are worthy of attention on this respect, as only 52.1% (37 out of 71) of the specimens were detected. False-negative result obtained using the MBU product could be associated with ineffective identification of certain genetic variants of the virus. However, despite the clear limitations, quite paradoxically the same assay showed the highest overall accuracy (99.87%), due to the higher specificity and to the much greater number of HIV-negative samples in the study population. This should be considered carefully when evaluating assay performance that are exclusively based on routine testing, being HIV-1 negative samples the majority in any setting.

As for specificity, according to the manufacturers’ specifications all the test systems here studied met the specified requirements. However according to a limited number of studies previously carried out in the Russian Federation, specificity varied considerably depending on the population tested. In the study by Sharipova et al. [11], the diagnostic specificity of test systems from Bio-Rad, Vector-Best, and Diagnostic Systems was investigated on 440 serum specimens from pregnant women and resulted as the same for all, up to 98.64% (97.06–99.37%). In our study, similar results were obtained for these test systems on a cohort of patients with potentially interfering conditions (99.00–99.83%). However, fundamentally different results were obtained with the Bio-Rad and especially Vector-Best test systems when the cohort of individuals at high risk of infection was tested. A significant drop in specificity in this case is of extreme interest, necessitating more research to identify its causes. A reduced specificity in populations at a high risk of acquiring HIV will lead to an increase in the number of confirmatory assays to be executed, (immunoblot and molecular biology for HIV-RNA), with a definite impact on costs and workflow, especially for HIV-RNA testing that is often carried out on a second specimen and in a different lab from the one where initial testing has been performed. On this, the convergence of the results obtained with initial positive and repeated positive sera was also quite different among the five assays. The test system manufactured by Abbott showed the best characteristics and 100% reproducibility, when tested for the reliability of negative and positive results and incidence of repeated positive results, while the lowest values of signal-to-noise ratio and, hence, the least difference between the initial positive and repeated positive results (10.49%) was demonstrated by the Vector-Best product. The sum of those performance characteristics determines not only the specificity of test systems, but also time and financial costs incurred by the laboratory which performs screening. This is an important characteristic, since it demonstrates test reliability and impacts the testing workflow by having a definite influence on both the number of assays and the time needed to get the final result.

The epidemiological findings did unveil some interesting aspects. The prevalence data we obtained, though on a sample size that cannot be considered as representative of the whole country, deserve some comments, confirm previous reports and highlight several needs [25]. More specifically, a 0.4% prevalence in the general population, and the significantly higher prevalence in people aged less than 40 years is in accord with previous data from Russia as well as from other countries [4, 26]. The huge prevalence among drug users and patients attending dermo-venereal clinics was not surprising and confirms the continuous spread of HIV by parenteral and sexual routes [26]. The latter is of high relevance, and allows to sustain the indication for HIV testing for all patients reporting to those centers on the double purpose of finding a substantial number of “hidden” infections and starting timely the contact tracing to limit the further spread.

An element of novelty compared to previous HIV serological surveys carried out in the Russian Federation is the assessment of time from infection through the determination of HIV antibody avidity. This is one of the several laboratory methods aimed to identify recent HIV infection by the so-called RITA that has been extensively dealt with by WHO [27]. According to those recommendations, those methods are “able to classify HIV infections in a population according to whether or not they were acquired in the recent past generally within 4 to 12 months [27]. The HIV avidity procedure we have employed has been demonstrated a high accuracy in discriminating recent from established HIV infections with a threshold of recency of 6 months from infection [13, 14, 28, 29] with a very low false recency rate [30]. In this study, the overall frequency of recent infections (5.4%) was aligned to a recent observation from Spain (5%) [31] but quite low compared to a recent report from UK that indicates a frequency of recent infections among newly diagnosed HIV cases of 8–19% [29]. The main reason for that is that avidity was strikingly higher in the general population (20%), where most of the tested population derived from, compared to only 4.2% among subjects belonging to high risk groups. A possible explanation of the latter is that 262 out of 391 (67%) of subjects at high risk have been enrolled from an AIDS Center and quite possibly a majority of patients attending that Center are not observed for the first time but followed up after a previous HIV diagnosis. On the other side, if 1/5 of the newly diagnosed cases in the general population have been infected over the last 6 months – plus the recent HIV in Fiebig II stage already described - the incidence in Russia may also be higher than previously reported.

From a diagnostic viewpoint, this study is the only direct comparison of the 4th generation serological test systems for screening for HIV infection used in the Russian Federation to be conducted on an extensive number of samples. Furthermore, the inclusion of a variety of different specimens and panels allowed for a more accurate evaluation of the assays performance. While in general terms all five assays considered appear at least adequate for first-line testing, some differences emerged both on sensitivity and specificity. The former appears to be affected by the time of infection – a false negative and different performances on seroconversion panels – and mostly by viral diversity, which shall rise concerns due to the increase of HIV viral diversity in Russia, as well as in most European countries and regions. On the other side, while assay specificity was good to excellent by all assays considered, on selected populations at high risk of infection we found a very low specificity by two assays. While the high prevalence of HIV infections in those populations will tamper the effect of this low specificity on the positive predictive value of the initial assay, the need of unnecessary test repetitions and confirmatory assays should be considered.

### Limitations

There were a number of limitations to our study. First, the ratio of recruited groups of patients in the study, belonging to the general population and high risk groups, did not match the characteristics of the epidemic in the country. For this study, we did not collect epidemiological information regarding the supposed route of transmission. In particular, we do not know how many identified HIV-positive men who attended dermo-venereal clinics had sex with men. Therefore, it is impossible to unambiguously extrapolate the characteristics of immunoassays obtained in this work for their routine use. The study also did not involve virus subtyping in the positive samples found.

## Conclusion

Obtaining a comprehensive picture of HIV assay performance in different populations and at different stages on the infection shall enable to plan for the optimal testing strategies that need to combine a good diagnostic accuracy and operational characteristics with the highest possible sensitivity, being the finding of HIV positive cases the goal for any screening program.

## Supplementary Information

**Additional file 1: Supplemental Figure 1.** Distribution of sample-to-cutoff (S/CO) ratios by the two 4th generation assays yielding the most similar S/CO mean on negative samples (Abbott and MBU): the distribution by the latter is skewed to the right, resulting in a much lower standard deviation (SD) ratio of the difference between the assay cutoff and the mean S/CO. X axis = number of results; Y axis = S/CO values.

**Additional file 2: Supplemental Figure 2.** Scheme of testing algorithm in the study.

**Additional file 3: Supplementary Figure 3.** Scheme of confirmatory HIV testing in the study.

**Additional file 4: Supplemental Table 1.** Incidence of initial and repeated positive results of clinical specimen testing.

**Additional file 5: Supplemental Table 2.** Performance of five HIV 4th generation immunoassays on 71 members of a HIV-1 p24 Viral Diversity panel. Genotyping = HIV-1 group, subtype or recombinant form; N = number of samples; + = positive result.

## Data Availability

The dataset from this study is available on request, provided that a clear statement on the purpose of the request will be provided.

## Abbreviations

Ab: Antibody
Ag: Antigen
AU: Autoimmune diseases
CI: Confidence interval
CIS: Commonwealth of Independent States
CLIA: Chemiluminescence Immunoassay
CRIE: Central Research Institute of Epidemiology
DA: Drug users
DS: Diagnostic Systems
ELISA: Enzyme-linked immunosorbent assay
FDA: U.S. Food and Drug Administration
GP: General population
HD: Hemodialysis patients
HIV: Human immunodeficiency virus
HR: High risk
IB: Immune blot
MBU: Medical Biological Union
PW: Pregnant women
RITA: Recent infection testing algorithm
RNA: Ribonucleic acid
RR: Repeat reactive
S/CO ratio: Signal to cutoff ratio
SD: Standard deviation
STD: Sexually transmitted diseases
UNAIDS: Joint United Nations Programme on HIV/AIDS
WHO: World Health Organization

## References

[1] UNAIDS. 90–90-90 An ambitious treatment target to help end the AIDS epidemic: UNAIDS / JC2684. Joint United Nations Programme on HIV/AIDS (UNAIDS); 2014. https://www.unaids.org/sites/default/files/media_asset/90-90-90_en.pdf.

[2] UNAIDS data 2018. Document is Available at http://www.unaids.org/sites/default/files/media_asset/unaids-data-2018_en.pdf. Accessed 23 June 2019.

[3] Anon. SanPiN 3.1.5.2826–10 Prevention of HIV infection (version dated 21.07.2016).

[4] Anon. HIV infection, Information Bulletin No. 43, Federal Scientific and Methodological Center for Prevention and Control of AIDS, Federal Budgetary Scientific Institution, Central Research Institute of Epidemiology, Federal Service for Surveillance on Consumer Rights Protection and Human Wellbeing (Rospotrebnadzor), 2018.

[5] Simon V, Ho DD, Quarraisha AK (2006). HIV/AIDS epidemiology, pathogenesis, prevention, and treatment. Lancet.

[6] Centers for Disease Control and Prevention and Association of Public Health Laboratories. Laboratory Testing for the Diagnosis of HIV Infection: Updated Recommendations. Available at http://stacks.cdc.gov/view/cdc/23447. Published June 27, 2014. Accessed 24 June 2019.

[7] Hemelaar J (2013). Implications of HIV diversity for the HIV-1 pandemic. J Inf Secur.

[8] Baranova EN (2009). The ability of modern test systems to confirm early HIV infection. Issues Virol.

[9] Ivanova NI, Peksheva OY (2009). The experience of HIV marker detection using a new ELISA diagnostic test system DS-EIA-HIV-AB/AG-SPECTRUM in the laboratories of the centers of AIDS prevention and control of the Privolzhsky Federal District. Clin Lab Diagn.

[10] Raspopina IV, Pankova LV, Kozhevnikova IV (2013). The experience of HIV marker detection using a new ELISA diagnostic test Invitrologic HIV 1,2-AG/AB. Bull Chelyabinsk Reg Clin Hosp.

[11] Sharipova IN (2015). Comparative study of the specificity of the test systems for the diagnosis of HIV infection in the serum specimens from pregnant women. Clin Lab Diagn.

[12] Lisitsyna ZN (2017). Immune tests and diagnosis of acute HIV infection. HIV Infect Immune Suppression.

[13] Suligoi B (2011). Avidity index for anti-HIV antibodies: comparison between third- and fourth-generation automated immunoassays. J Clin Microbiol.

[14] Suligoi B (2017). HIV avidity index performance using a modified fourth-generation immunoassay to detect recent HIV infections. Clin Chem Lab Med.

[15] Fiebig EW (2003). Dynamics of HIV viremia and antibody seroconversion in plasma donors: implications for diagnosis and staging of primary HIV infection. AIDS.

[16] Branson BM. Human immunodeficiency virus diagnostics: current recommendations and opportunities for improvement. Infect Dis Clin N Am. 2019. 10.1016/j.idc.2019.04.001. 10.1016/j.idc.2019.04.001 31239094

[17] Anon. HIV assays: laboratory performance and other operational characteristics: rapid diagnostic tests (combined detection of HIV-1/2 antibodies and discriminatory detection of HIV-1 and HIV-2 antibodies): report 18. World Health Organization; 2015. ISBN 978 92 4 150811 7.

[18] Chaves P (2011). Evaluation of the performance of the Abbott ARCHITECT HIV Ag/Ab combo assay. J Clin Virol.

[19] Mitchell EO (2013). Performance comparison of the 4th generation bio-rad laboratories GS HIV combo Ag/Ab EIA on the EVOLIS™ automated system versus Abbott ARCHITECT HIV Ag/Ab combo, Ortho anti-HIV 1+2 EIA on Vitros ECi and Siemens HIV-1/O/2 enhanced on Advia centaur. J Clin Virol.

[20] Lapovok IA (2017). Molecular epidemiological analysis of HIV-1 variants circulating in Russia in 1987—2015. Ther Arch.

[21] Murzakova A (2019). Molecular epidemiology of HIV-1 subtype G in the Russian Federation. Viruses.

[22] Gaudy C (2004). Subtype B human immunodeficiency virus (HIV) type 1 mutant that escapes detection in a fourth generation immunoassay for HIV infection. J Clin Microbiol.

[23] Ly TD (2012). The variable sensitivity of HIV Ag/Ab combination assays in the detection of p24 Ag according to genotype could compromise the diagnosis of early HIV infection. J Clin Virol.

[24] Qiu X (2017). Comparative evaluation of three FDA-approved HIV Ag/Ab combination tests using a genetically diverse HIV panel and diagnostic specimens. J Clin Virol.

[25] Leeflang MMG, Allerberger F (2019). Sample size calculations for diagnostic studies. Clin Microbiol Infect.

[26] European Centre for Disease Prevention and Control, WHO Regional Office for Europe (2018). HIV/AIDS surveillance in Europe 2018–2017 data.

[27] UNAIDS/WHO Working Group on Global HIV/AIDS and STI Surveillance. When and how to use assays for recent infection to estimate HIV incidence at a population level: World Health Organization; 2011. https://www.who.int/hiv/pub/surveillance/sti_surveillance/en/. Accessed 27 June 2019. ISBN 978 92 4 150167 5.

[28] Sweeting MJ (2010). Estimating the distribution of the window period for recent HIV infections: a comparison of statistical methods. Stat Med.

[29] Aghaizu A (2018). HIV incidence among sexual health clinic attendees in England: first estimates for black African heterosexuals using a biomarker, 2009-2013. PLoS One.

[30] Kassanjee R (2016). Viral load criteria and threshold optimization to improve HIV incidence assay characteristics. AIDS.

[31] Nicolàs D (2019). Epidemiological changes of acute/recent human immunodeficiency virus type 1 infection in Barcelona, Spain (1997/2015): a prospective cohort study. Clin Microbiol Infect.

