# Supplementary figures and images for: Serological diagnosis and prevalence of HIV-1 infection in Russian metropolitan areas
Source: BMC Infect Dis. 2021 Jan 7;21:24. doi: 10.1186/s12879-020-05695-z (PMC7791727; doi:10.1186/s12879-020-05695-z)

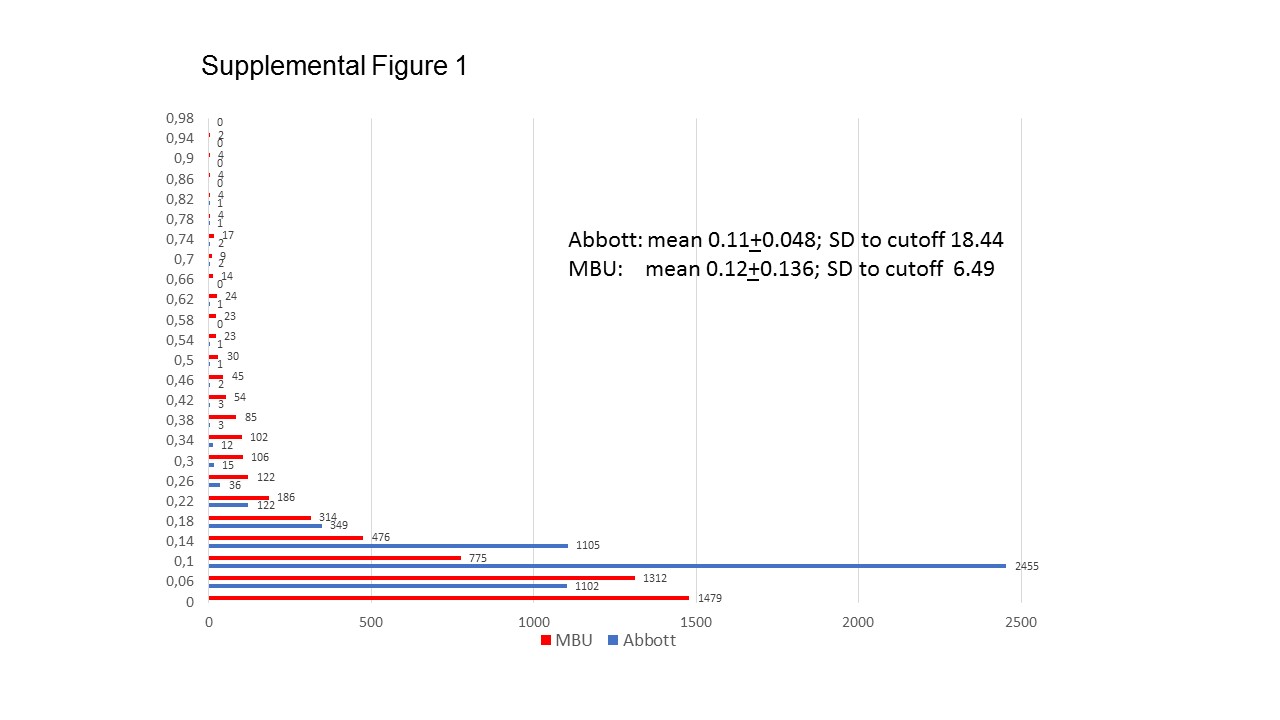

Supplement: Supplementary file 1 — Additional file 1: Supplemental Figure 1. Distribution of sample-to-cutoff (S/CO) ratios by the two 4th generation assays yielding the most similar S/CO mean on negative samples (Abbott and MBU): the distribution by the latter is skewed to the right, resulting in a much lower standard deviation (SD) ratio of the difference between the assay cutoff and the mean S/CO. X axis = number of results; Y axis = S/CO values. [file 12879_2020_5695_MOESM1_ESM.jpg]

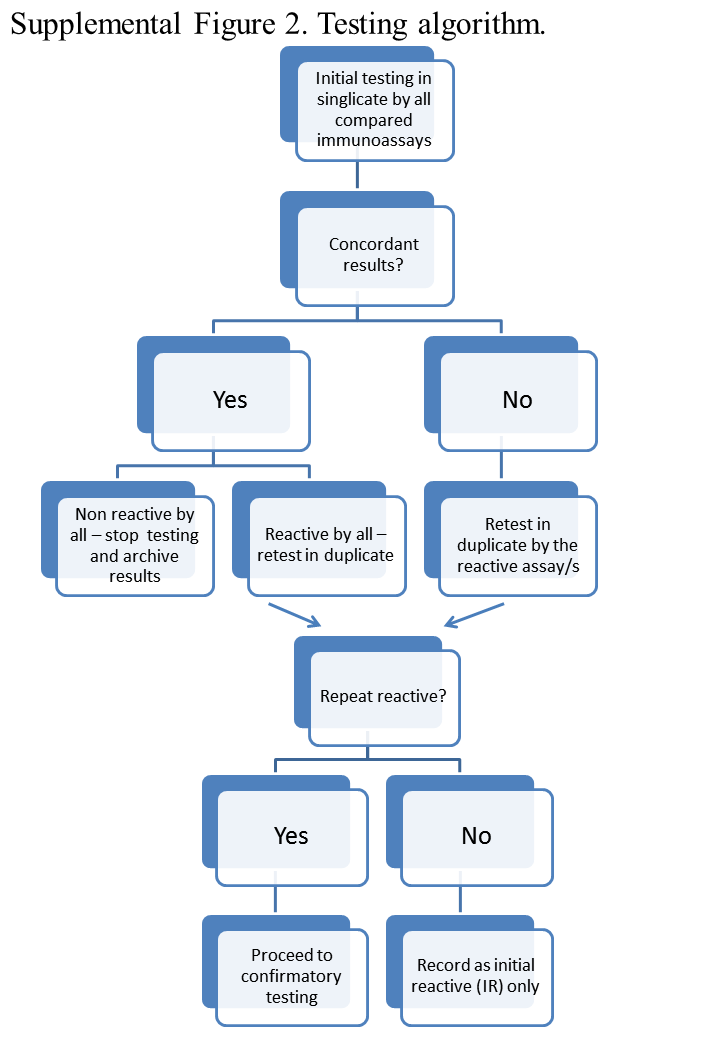

Supplement: Supplementary file 2 — Additional file 2: Supplemental Figure 2. Scheme of testing algorithm in the study. [file 12879_2020_5695_MOESM2_ESM.tif]

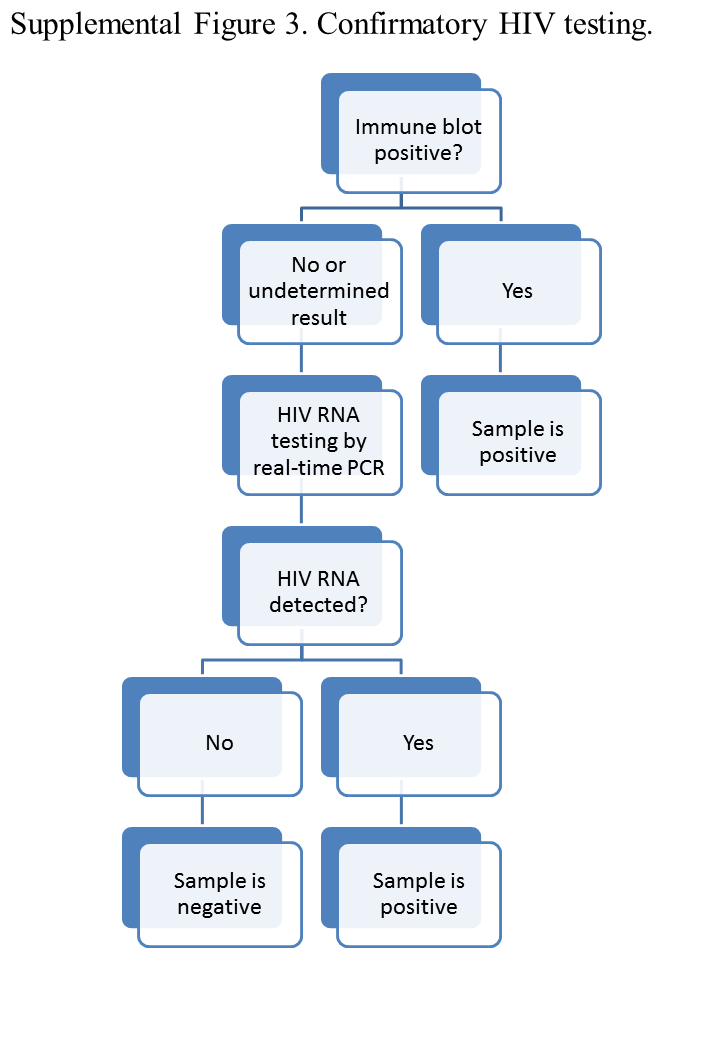

Supplement: Supplementary file 3 — Additional file 3: Supplementary Figure 3. Scheme of confirmatory HIV testing in the study. [file 12879_2020_5695_MOESM3_ESM.tif]
