# Supplementary material for: Serological diagnosis and prevalence of HIV-1 infection in Russian metropolitan areas
Source: BMC Infect Dis. 2021 Jan 7;21:24. doi: 10.1186/s12879-020-05695-z (PMC7791727; doi:10.1186/s12879-020-05695-z)
Supplement: Supplementary file 4 — Additional file 4: Supplemental Table 1. Incidence of initial and repeated positive results of clinical specimen testing. [file 12879_2020_5695_MOESM4_ESM.docx]

Supplemental Table 1. Incidence of initial and repeated positive results of clinical specimen testing

| **Cohort** | **Parameter** | **Abbott** | **Bio-Rad** | **Vector-Best** | **DS** | **MBU** |
| --- | --- | --- | --- | --- | --- | --- |
| General population | Initial positive | 0.56% (25/4452) [0.36 - 0.83] | 0.76% (34/4452) [0.53 - 1.07] | 0.79% (35/4452) [0.55 - 1.09] | 1.08% (48/4452) [0.80 - 1.43] | 0.85% (38/4452) [0.60 - 1.17] |
|  | Repeated positive | 0.56% (25/4452) [0.36 - 0.83] | 0.52% (23/4452) [0.33 - 0.77] | 0.47% (21/4452) [0.29 - 0.72] | 0.63% (28/4452) [0.42 - 0.91] | 0.47% (21/4452) [0.29 - 0.72] |
|  | Difference between initial and repeated positive | 0 (0%) | 11 (32.35%) | 14 (40.00%) | 20 (41.67%) | 17 (44.74%) |
| Individuals at high risk or with potentially interfering conditions | Initial positive | 20.20% (200/990) [17.74 - 22.84] | 21.52% (213/990) [18.99 - 24.21] | 23.43% (232/990) [20.83 - 26.20] | 20.71% (205/990) [18.22 - 23.37] | 20.51% (203/990) [18.03 - 23.16] |
|  | Repeated positive | 20.20% (200/990) [17.74 - 22.84] | 21.11% (209/990) [18.61 - 23.79] | 22.02% (218/990) [19.47 - 24.73] | 20.30% (201/990) [17.84 - 22.95] | 20.30% (201/990) [17.84 - 22.95] |
|  | Difference between initial and repeated positive | 0 (0%) | 4 (1.88%) | 14 (6.04%) | 4 (1.95%) | 2 (0.99%) |
| Total | Initial positive | 4.13% (225/5442) [3.62 - 4.70] | 4.54% (247/5442) [4.00 - 5.13] | 4.91% (267/5442) [4.35 - 5.51] | 4.65% (253/5442) [4.10 - 5.24] | 4.43% (241/5442) [3.90 - 5.01] |
|  | Repeated positive | 4.13% (225/5442) [3.62 - 4.70] | 4.26% (232/5442) [3.74 - 4.83] | 4.39% (239/5442) [3.86 - 4.97] | 4.21% (229/5442) [3.69 - 4.78] | 4.08% (222/5442) [3.57 - 4.64] |
|  | Difference between initial and repeated positive | 0 (0%) | 15 (6.07%) | 28 (10.49%) | 24 (9.49%) | 19 (7.88%) |
