# Supplementary material for: Serological diagnosis and prevalence of HIV-1 infection in Russian metropolitan areas
Source: BMC Infect Dis. 2021 Jan 7;21:24. doi: 10.1186/s12879-020-05695-z (PMC7791727; doi:10.1186/s12879-020-05695-z)
Supplement: Supplementary file 5 — Additional file 5: Supplemental Table 2. Performance of five HIV 4th generation immunoassays on 71 members of a HIV-1 p24 Viral Diversity panel. Genotyping = HIV-1 group, subtype or recombinant form; N = number of samples; + = positive result. [file 12879_2020_5695_MOESM5_ESM.docx]

Supplemental Table 2. Performance of five HIV 4^th^ generation immunoassays on 71 members of a HIV-1 p24 Viral Diversity panel. Genotyping= HIV-1 group, subtype or recombinant form; N= number of samples; += positive result

| Genotyping | N | Abbott+ | BioRad+ | Diagnostic Systems+ | Vector-Best+ | Medical Biological Unit+ |
| --- | --- | --- | --- | --- | --- | --- |
| A | 4 | 4 | 4 | 3 | 4 | 2 |
| B | 11 | 11 | 11 | 9 | 11 | 7 |
| C | 8 | 8 | 8 | 8 | 8 | 5 |
| D | 6 | 6 | 6 | 6 | 6 | 3 |
| F | 6 | 6 | 6 | 5 | 6 | 5 |
| G | 3 | 3 | 3 | 2 | 3 | 2 |
| H | 2 | 2 | 2 | 2 | 1 | 1 |
| J | 1 | 1 | 1 | 1 | 1 | 1 |
| CRF_01 | 9 | 9 | 9 | 8 | 9 | 2 |
| CRF_02 | 5 | 5 | 5 | 5 | 3 | 5 |
| CRF_06 | 2 | 2 | 2 | 2 | 1 | 1 |
| CRF_11 | 1 | 1 | 1 | 0 | 1 | 0 |
| URF_AB | 1 | 1 | 1 | 1 | 1 | 1 |
| URF_AG | 1 | 1 | 1 | 1 | 1 | 1 |
| Group O | 8 | 8 | 0 | 0 | 0 | 1 |
| Group N | 2 | 2 | 0 | 1 | 0 | 0 |
| Group P | 1 | 1 | 1 | 0 | 0 | 0 |
| Total | 71 | 71 | 61 | 54 | 56 | 37 |
